# Supplementary material for: An allosteric inhibitor of Mycobacterium tuberculosis ArgJ: Implications to a novel combinatorial therapy
Source: EMBO Mol Med. 2018 Feb 26;10(4):e8038. doi: 10.15252/emmm.201708038 (PMC5887547; doi:10.15252/emmm.201708038)
Supplement: Supplementary file 1 — Appendix [file EMMM-10-e8038-s001.pdf]

Table of contents

Appendix figure files with legends:

Appendix Fig. S1 -- 2

Appendix Fig. S2 -- 4

Appendix Fig. S3 -- 6

Appendix Fig. S4 -- 8

Appendix Fig. S5 -- 10

Appendix Fig. S6 -- 12

Appendix Fig. S7 -- 13

Appendix Fig. S8 -- 14

Appendix Fig. S9 -- 16

Appendix Fig. S10 -- 18

Appendix Fig. S11 -- 20

Appendix Materials and Methods --- 22

Appendix Results and Discussion -- 31

Appendix Table S1 --- 36

Appendix Table S2 --- 37

Appendix Table S3 --- 39

Appendix Table S4 --- 40

Appendix Table S5 --- 41

Appendix Table S6 --- 44

Appendix Table S7 --- 45

References -- 47

Appendix Fig. S1

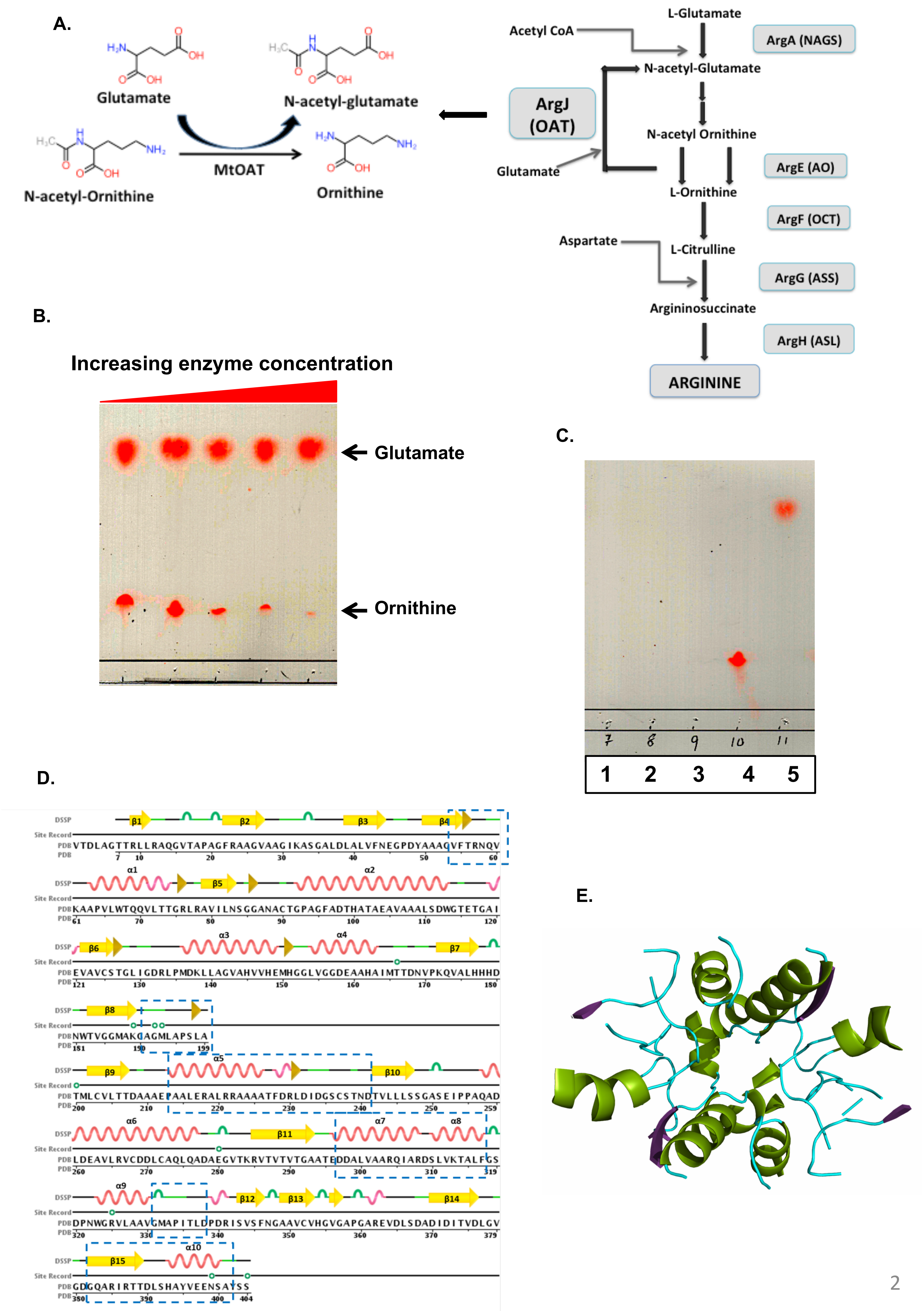

Appendix Fig. S1. Arginine biosynthesis pathway and characterization of *Mt*ArgJ enzyme. **(A)** Arginine biosynthesis pathway in detail (*Mt*ArgJ reaction shown separately). The large hydrophobic pocket of MtArgJ is shown. **(B)** TLC image for *Mt*ArgJ activity assay with varying enzyme concentration **(C)** TLC image for standards; 1- PRK, 2- SRB, 3- N-acetyl ornithine 4- Ornithine and 5- Glutamate **(D)** The pocket mapped on the secondary structures and the amino acid residues involved are enclosed in blue boxes (---) **(E)** Secondary structural features of the novel pocket.

Appendix Fig. S2

A.

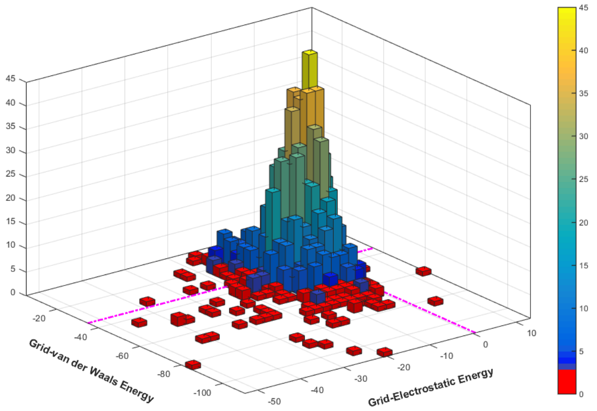

B.

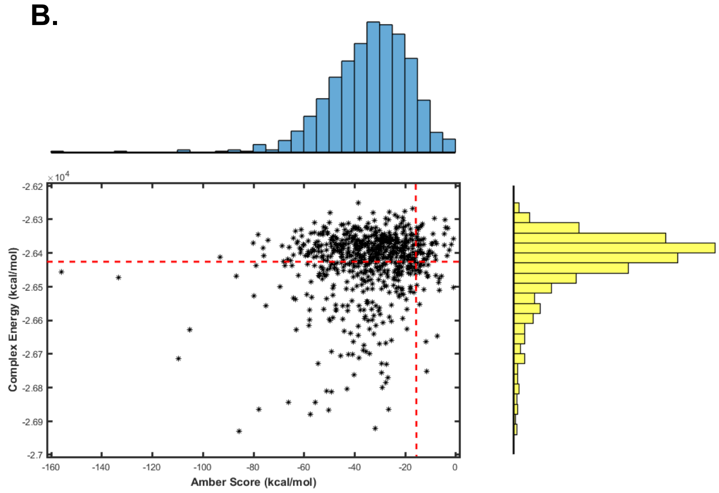

C.

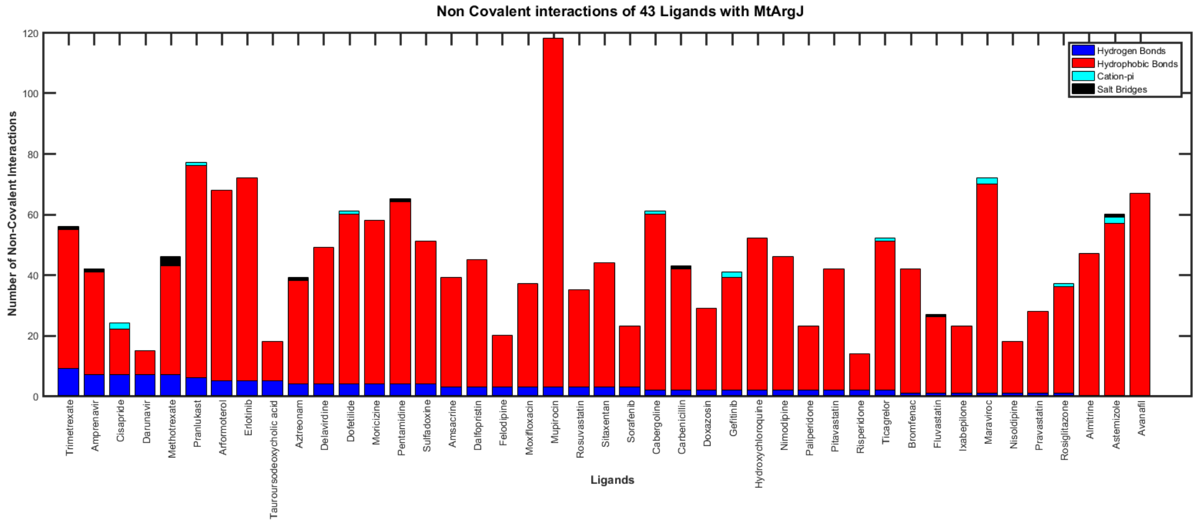

D.

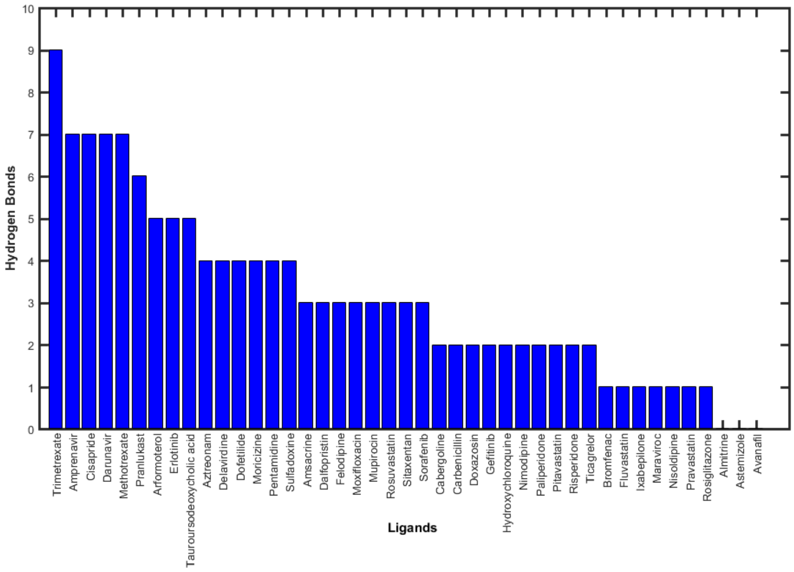

E.

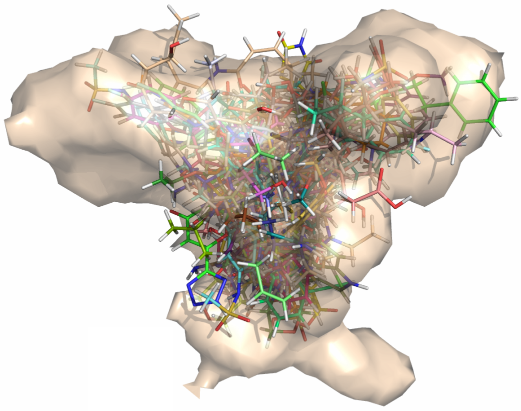

Appendix Fig. S2. Various filtering criterion implemented for virtual screening of potential hits against the large interface hydrophobic pocket in MtArgJ. (A) Bivariate histogram-based partitioning method applied for clustering 1340 ligands based on the electrostatic energies (ranging from 0.59 to -34.39 kcal/mol) and van der Waals energies (ranging between -5.43 and -109.25 kcal/mol). **(B)** Scatter plot showing the distribution of Amber scores for 738 receptor-ligand complexes and the dashed line in red marks the threshold set to filter the ligands based on the Amber score (-15.84 kcal/mol) and energy of MtArgJ-ANS complex (-26441.99 kcal/mol). **(C)** Number of non-covalent interactions formed between the major hydrophobic pocket and the compounds screened as computed *in silico* **(D)** Number of H-bonds formed between the compounds screened and the hydrophobic pocket **(E)** The receptor-ligand space of MtArgJ showing the docked conformations of final forty three ligands in the hydrophobic space.

Appendix Fig. S3

A.

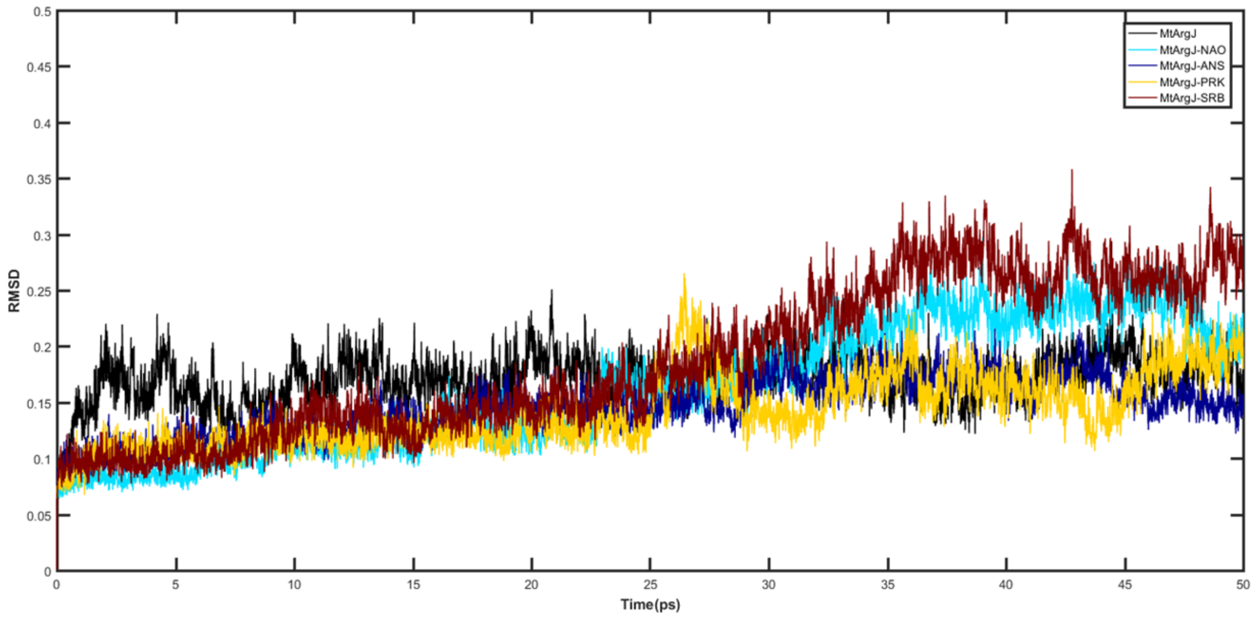

B.

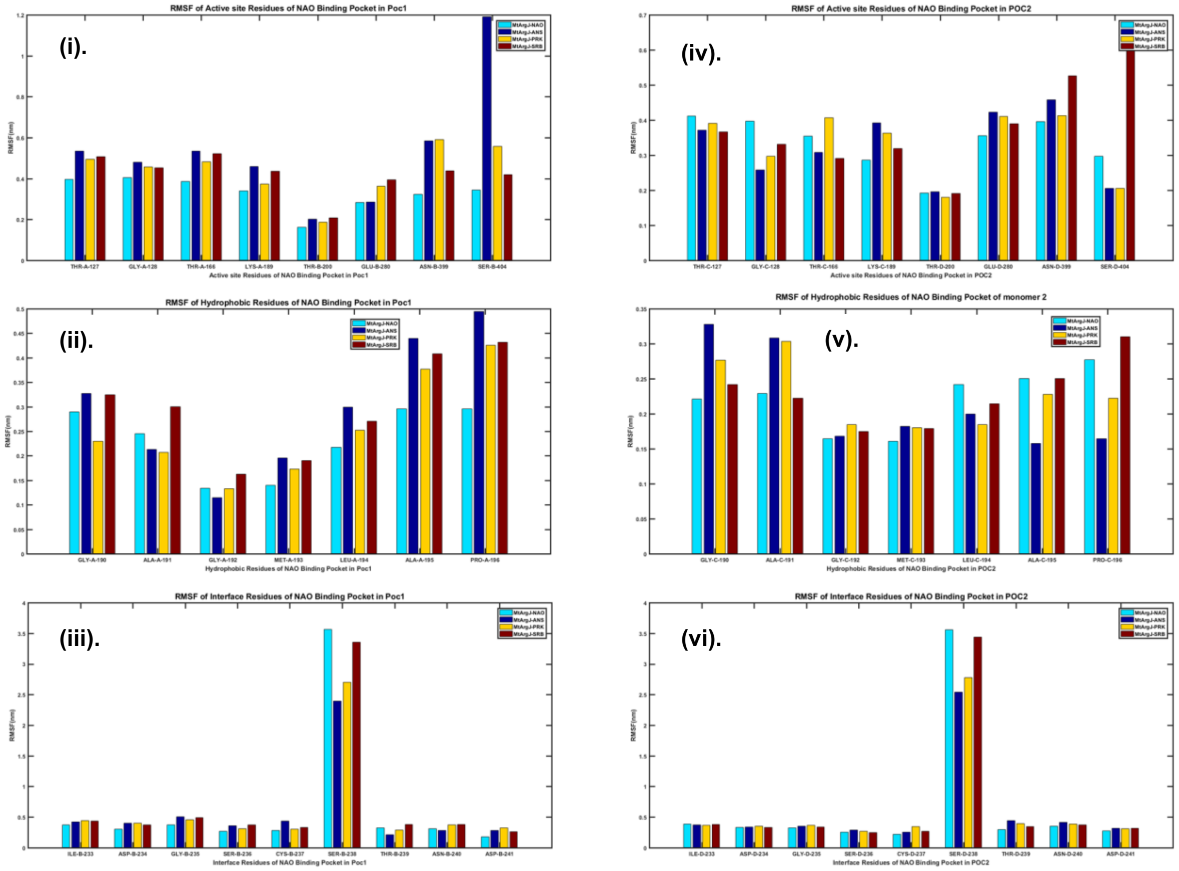

Appendix Fig. S3. RMSD and RMSF fluctuations. (A) RMSDs of *Mt*ArgJ in free (black) and complexed forms with only substrate (cyan), ANS (blue), pranlukast (yellow) and sorafenib (maroon) plotted as a function of time. **(B)** Root mean square fluctuations of amino acid residues in MtArgJ lining **(i)** the active site pocket in monomer -1 **(ii)** hydrophobic residues of active site pocket in monomer -1, **(iii)** interface residues of monomer -1 and large hydrophobic pocket, **(v)** the active site pocket in monomer -2 **(iv)** hydrophobic residues of active site pocket in monomer -2 and **(vi)** interface residues of monomer -2 and large hydrophobic pocket

Appendix Fig. S4

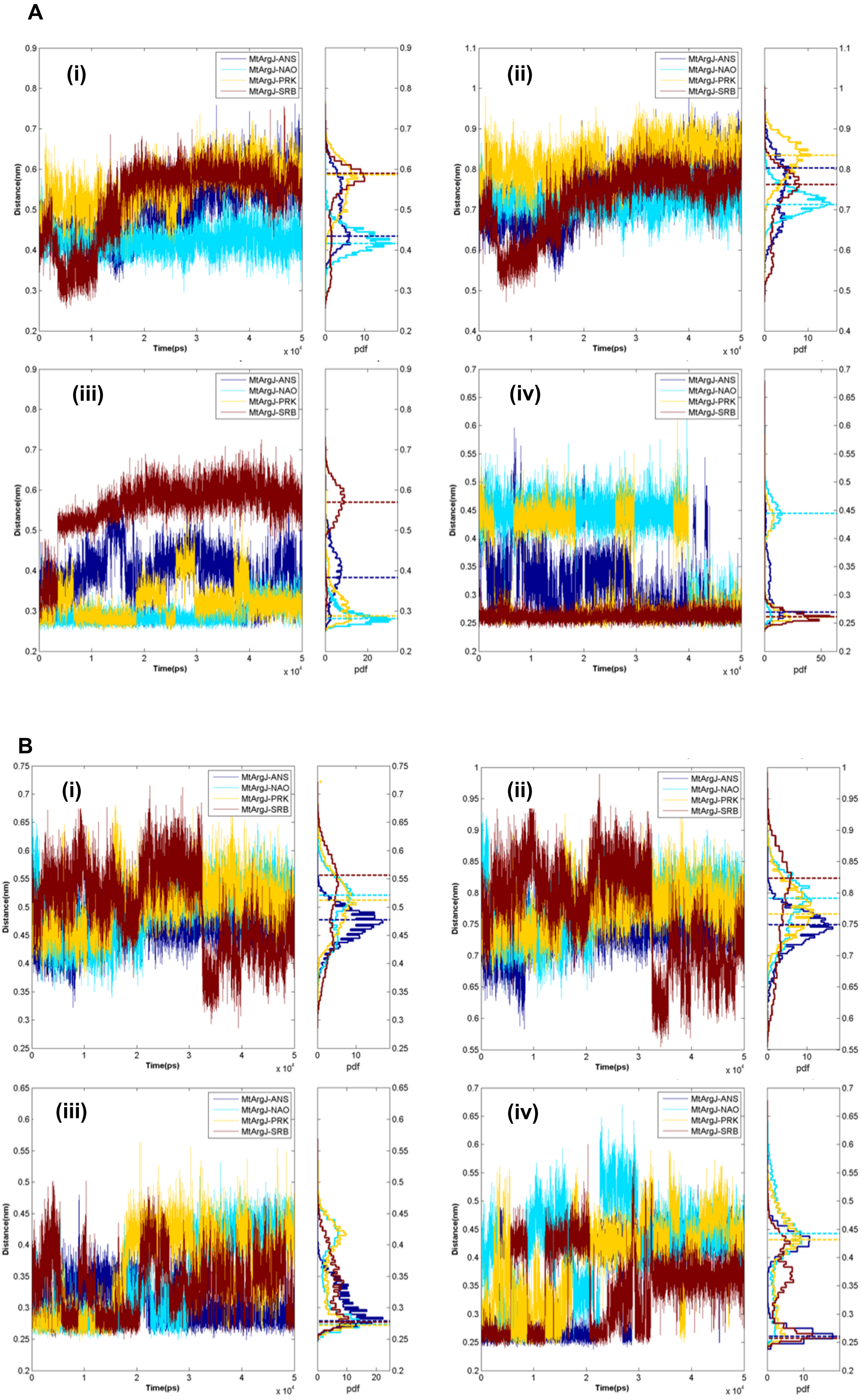

Appendix Fig. S4. Computational plots for distance between crucial residues. (A) Plots showing the distances between atoms of important active site residues in monomer-1 computed as a function of time for *Mt*ArgJ in complex with substrate (NAO), ANS, pranlukast (PRK) and sorafenib (SRB). Subpanels show distances computed between the atoms of the residues **(i)** Thr127(OG1) – Thr200(O), **(ii)** Gly128(N) – Thr200(O), **(iii)** Thr200(OG1) – Ser238(OG) and **(iv)** Ser238(OG) – Asp241(OD1) in all the four *Mt*ArgJ complexes from 50ns MD trajectory. **(B)** Plots showing the distances between atoms of important active site residues in monomer-2 computed as a function of time for *Mt*ArgJ in complex with substrate (NAO), ANS, pranlukast (PRK) and sorafenib (SRB). Subpanels show distances computed between the atoms of the residues **(i)** Thr127(OG1) – Thr200(O), **(ii)** Gly128(N) – Thr200(O), **(iii)** Thr200(OG1) – Ser238(OG) and **(iv)** Ser238(OG) – Asp241(OD1) in all the four MtArgJ complexes from 50ns MD trajectory

Appendix Fig. S5

A.

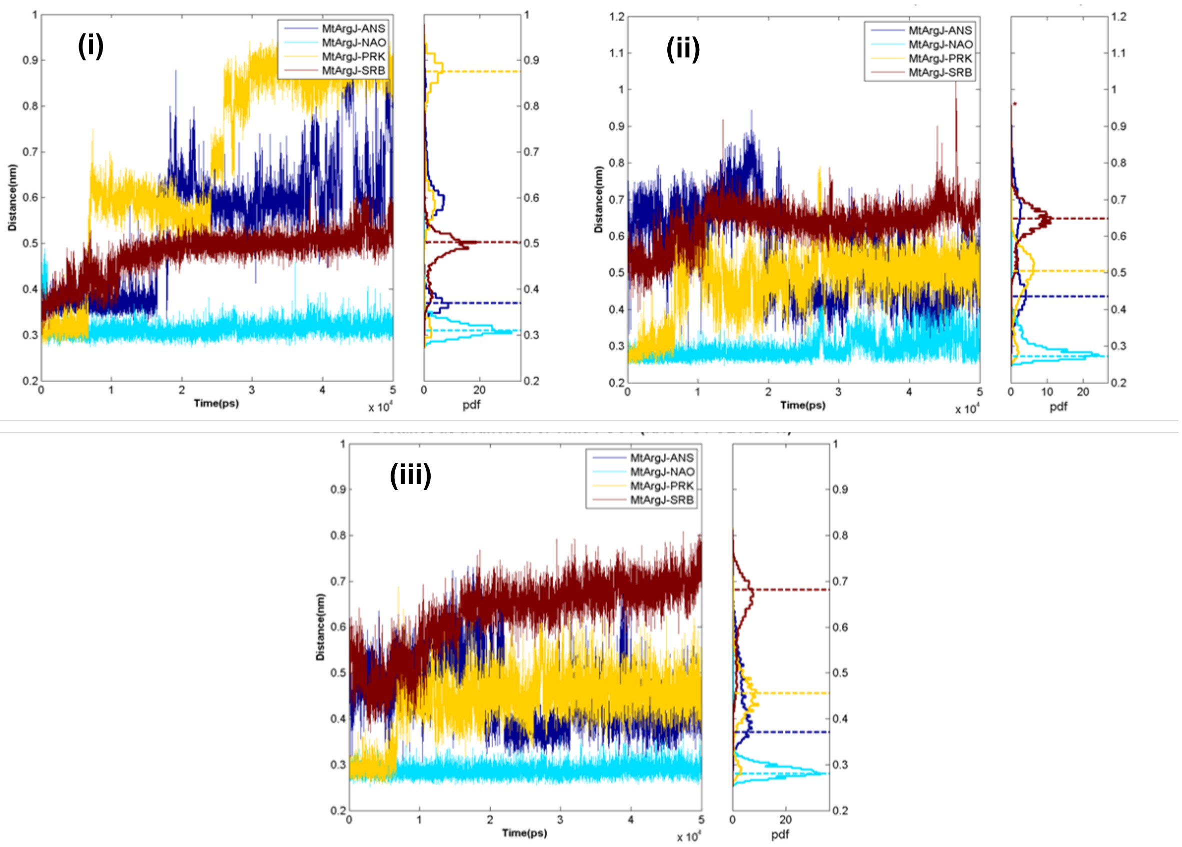

B.

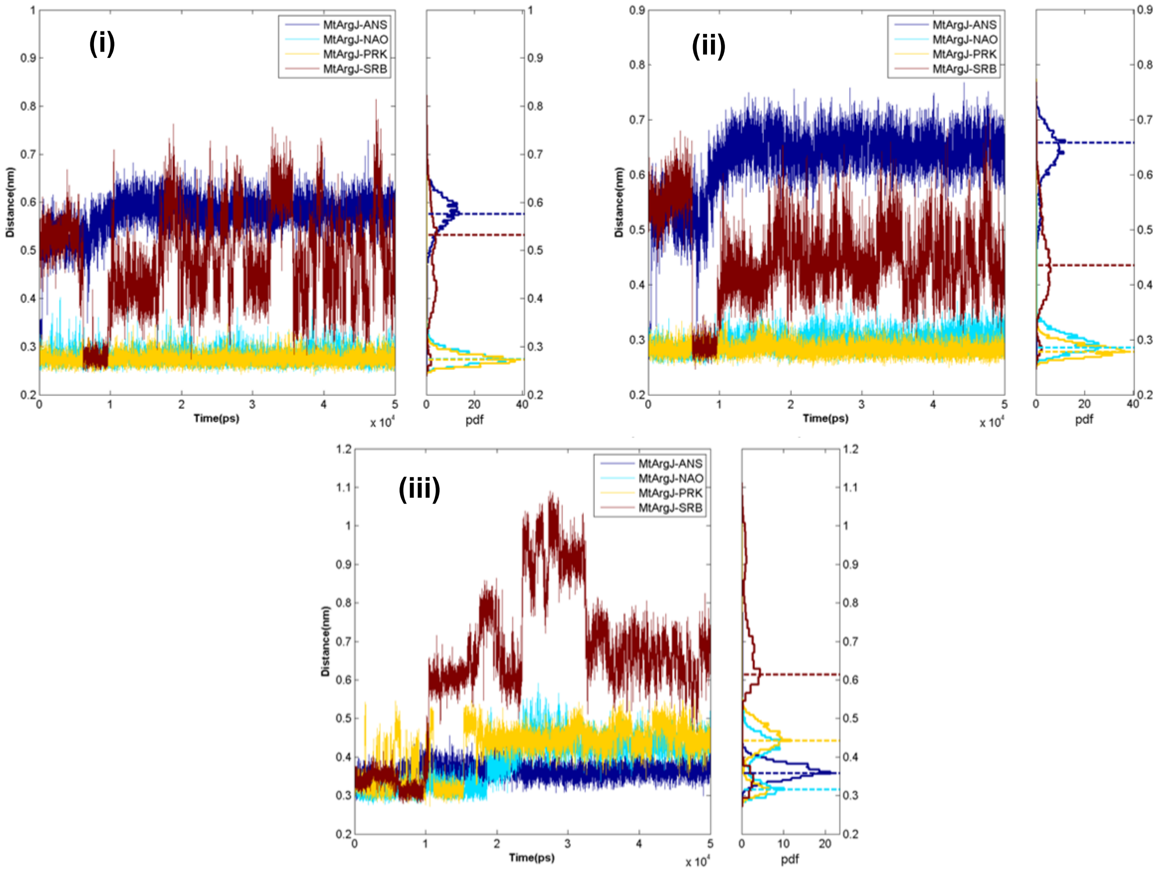

Appendix Fig. S5. Plots with residue distances as a function of time. (A) Plots showing the distances as a function of time computed between atoms of important active site residues and the substrate (NAO) in monomer-1 of MtArgJ bound to substrate (NAO) and with other three inhibitors viz., ANS, pranlukast (PRK) and sorafenib (SRB). Subpanels show distances computed between the atoms of the residues **(i)** NAO1(C1) – Thr200(OG1), (**ii)** NAO1(O1) – Thr127(OG1) and **(iii)** NAO1(O1) – Gly128(N) in all the four MtArgJ complexes from 50ns MD trajectory. **(B)** Plots showing the distances as a function of time computed between atoms of important active site residues and the substrate (NAO) in monomer-2 of MtArgJ bound to substrate (NAO) and with other three inhibitors viz., ANS, pranlukast (PRK) and sorafenib (SRB). Subpanels show distances computed between the atoms of the residues **(i)** NAO1(C1) – Thr200(OG1), (**ii)** NAO1(O1) – Thr127(OG1) and **(iii)** NAO1(O1) – Gly128(N) in all the four MtArgJ complexes from 50ns MD trajectory.

Appendix Fig. S6 ***Mt*ArgJ-ANS**

A.

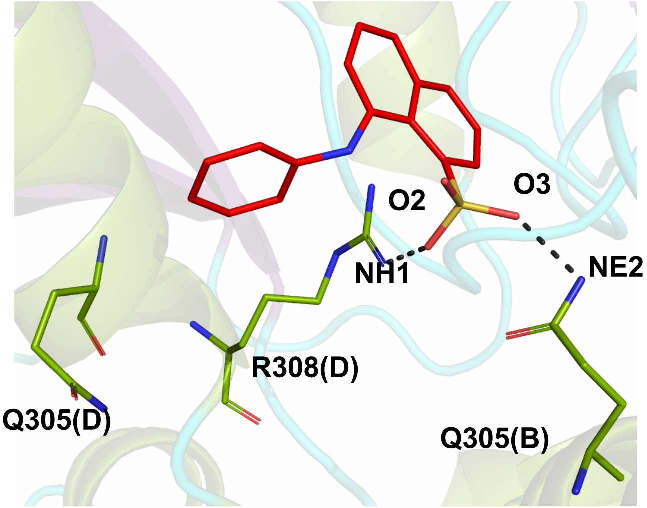

B.

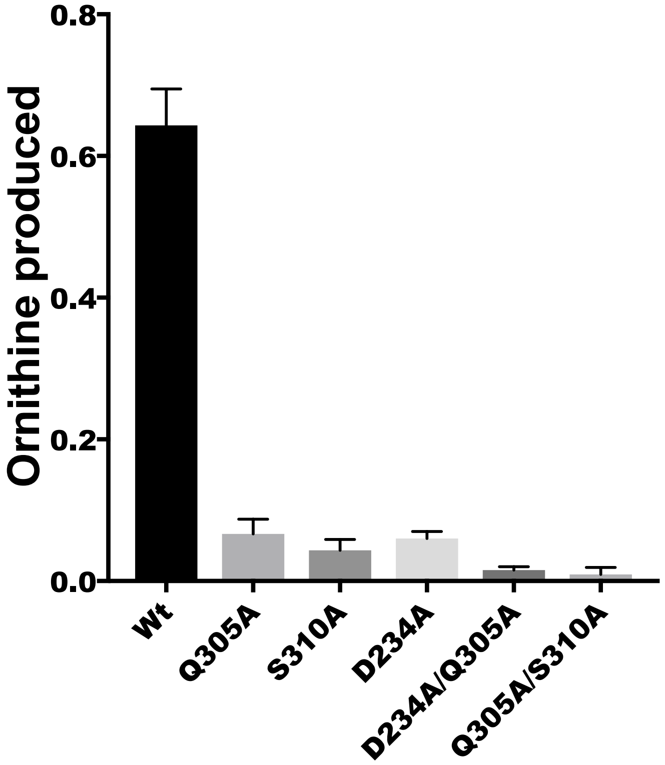

Appendix Fig. S6 A. Binding conformations of ANS in the allosteric pocket of *Mt*ArgJ. Hydrogen bond interactions of ANS (red) with Gln305 of B and D chains and Arg308 of D chain are depicted**.**

B. Based on MD simulations analysis, residue number Q^{305}, S^{310}, D^{234} were each mutated to alanine and double mutants of D^{234}/ Q^{305} and Q^{305}/ S^{310} were also generated (site directed mutagenesis) . The mutants were assayed for their enzymatic activity (substrate, N-acetyl ornithine was kept constant at 1mM) and were found to be devoid of enzymatic activity.

Appendix Fig. S7

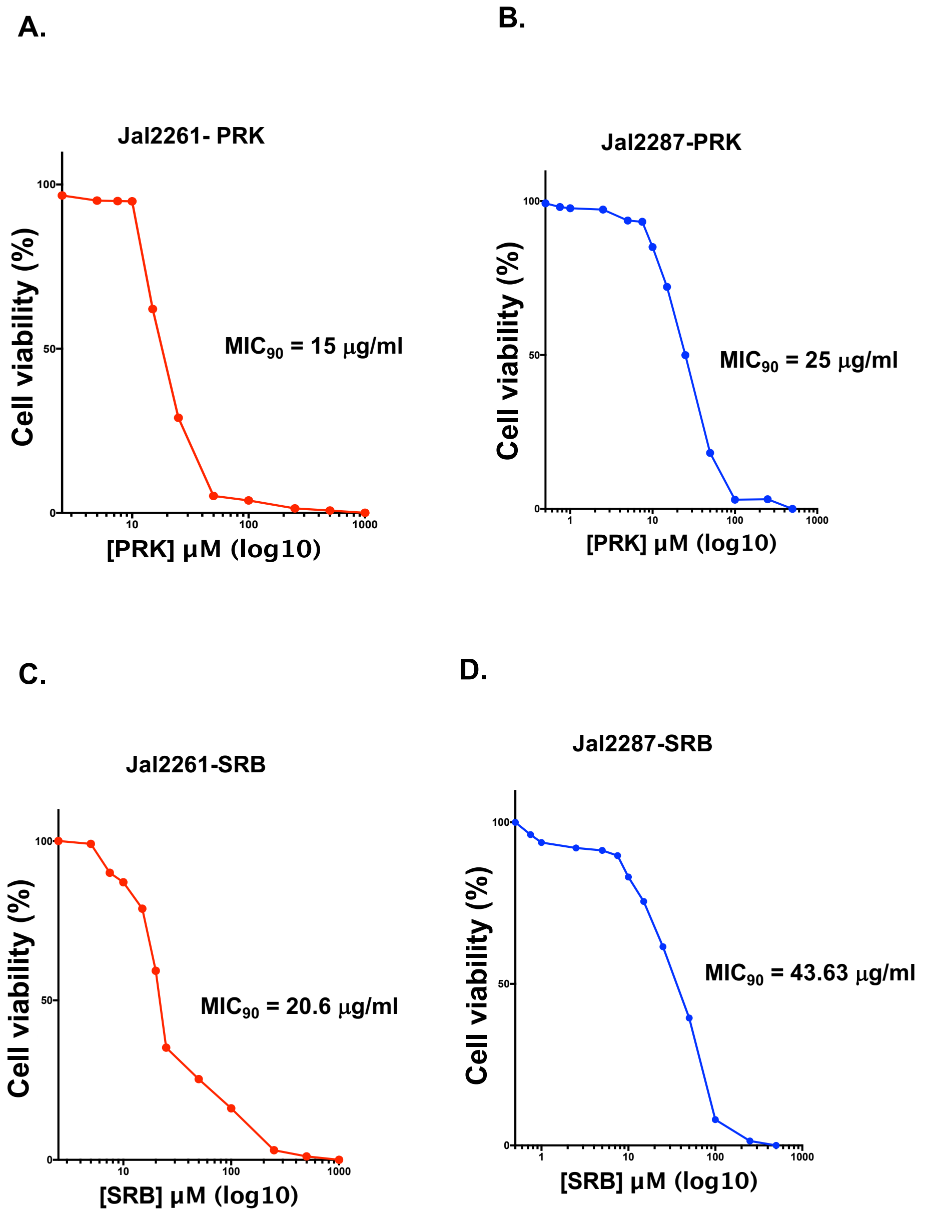

Appendix Fig. S7. Effect of PRK and SRB on the Multi drug resistant strains of *Mtb.* *Mycobacterium tuberculosis* MDR strains Jal 2261 and Jal 2287 were treated with varying concentration of inhibitors and cell viability was determined using Alamar blue assay. MIC was calculated by plotting cell viability (%) against increasing concentration of inhibitors: (**A**). Effect of PRK on Jal2261 (**B**). Effect of PRK on Jal2287 (**C**). Effect of SRB on Jal 2261 and **(D).** Effect of SRB on Jal2287 MDR strains.

Appendix Fig. S8

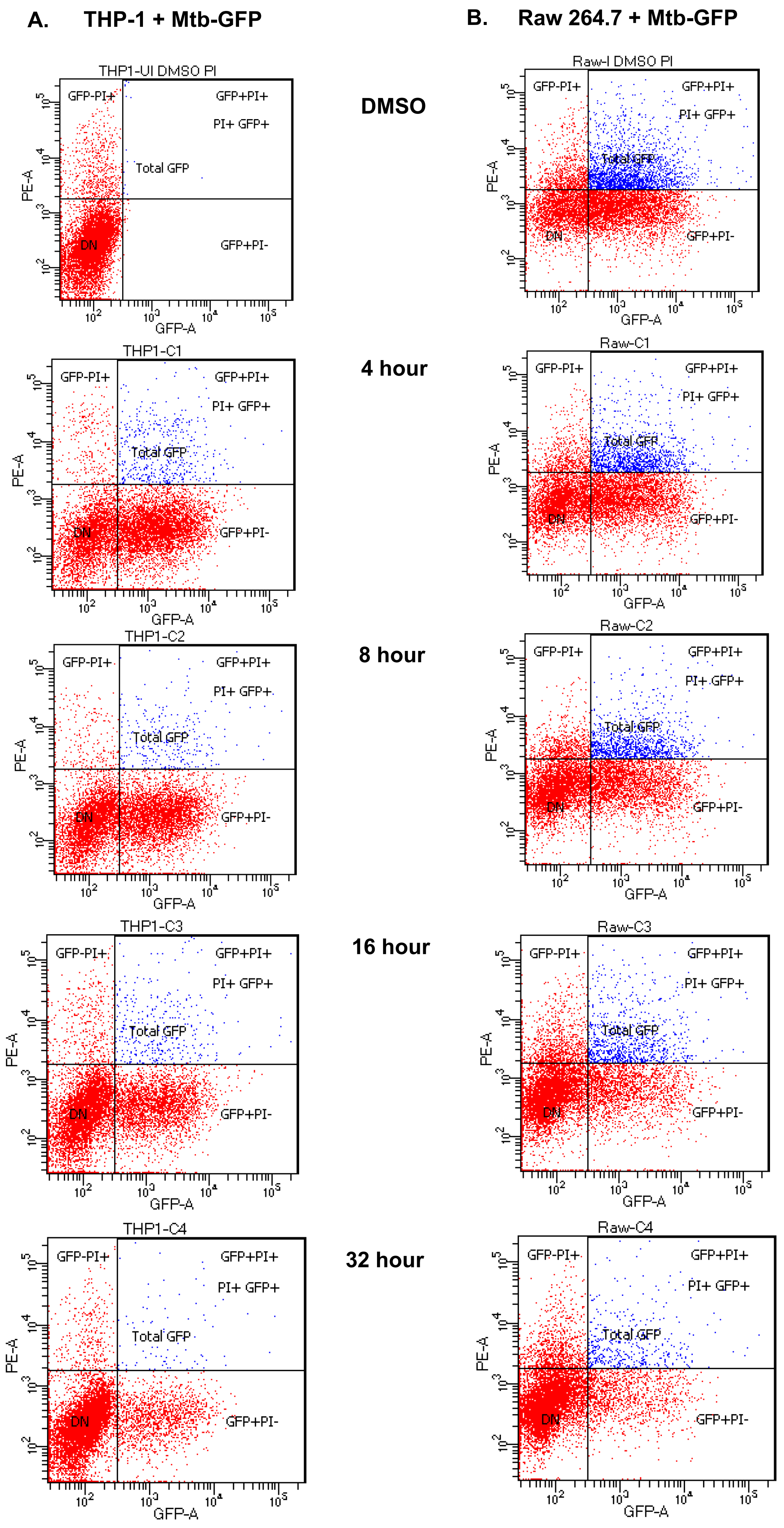

Appendix Fig. S8. Flow cytometry analysis of *Mtb* (H37Rv-GFP) infected macrophages. Determining the Mtb internalization upon infection and the effect of PRK treatment in reducing bacterial burden from infected macrophages (**A).** THP1 and **(B)** Raw264.7. Also, the FACS scatter graph shows the simultaneous detection of PI staining, which indicates the macrophage cell death associated with infection and consecutive drug treatment.

Appendix Fig. S9

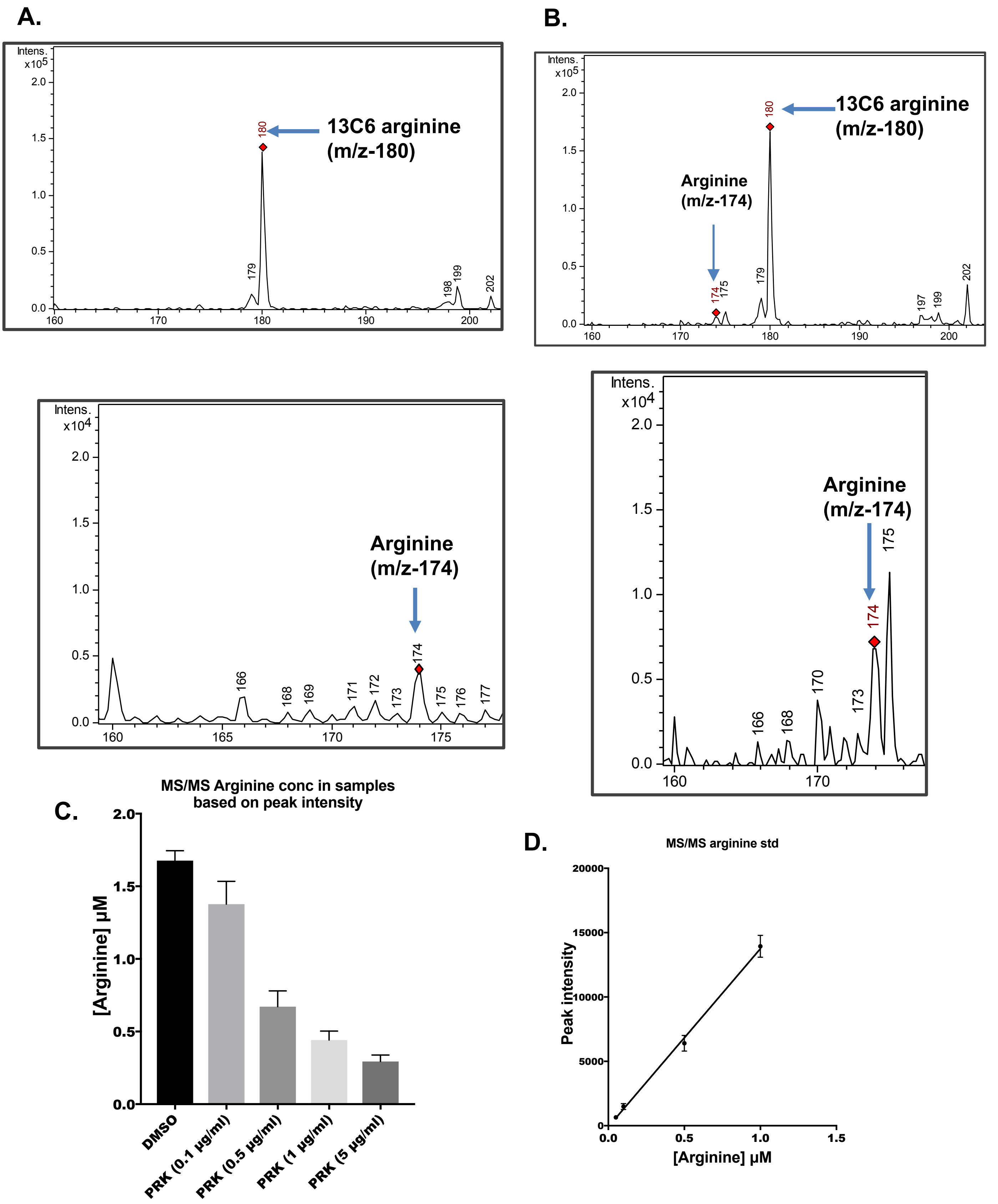

Appendix Fig. S9. Mass spectrometry analysis of *Mtb* arginine levels

ESI/MS analysis of the whole cell metabolites isolated from *Mtb* samples. Metabolite samples from **(a)** PRK treated *Mtb* cells (b) DMSO treated *Mtb* cells were subjected to ESI-MS analysis and arginine peak was identified as m/z 174. The peaks were compared and analyzed for arginine levels based on peak area (refer Fig 7b) and **(c)** intensity. The peak m/z 180 corresponds to the 13C6 labelled arginine as internal control. Notice peak m/z 174 in **(a)** and **(b)** lower panel for arginine. **(c)** Concentration of arginine in the metabolite isolated from PRK treated *Mtb* sample at four different PRK concentrations, was calculated by a standard arginine plot based on peak intensity, **(d)** Arginine standard were subjected to ESI analysis and peak intensity was plotted as a function of know arginine concentration.

Appendix Fig. S10

A. Control Mice (PBS treated)

Lungs

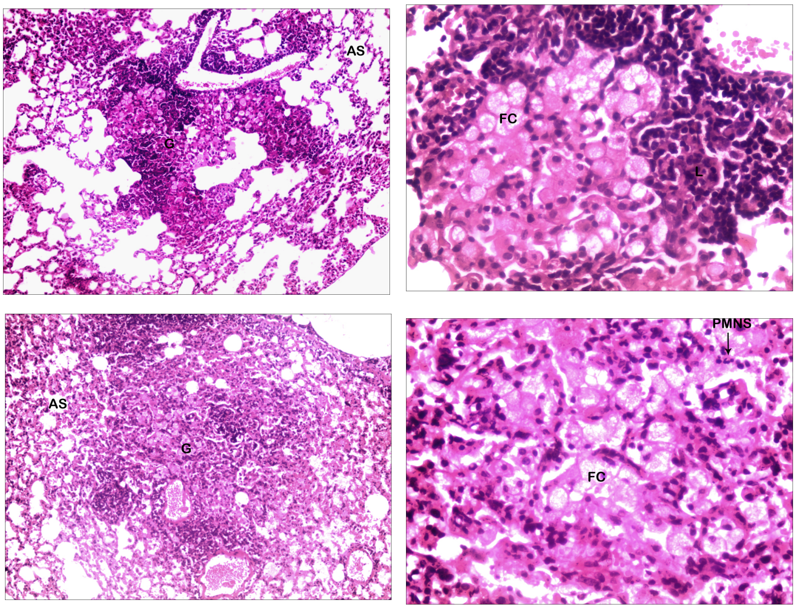

100x 400x

Spleen

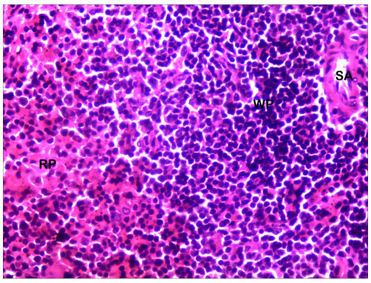

400x

Hepatic tissues

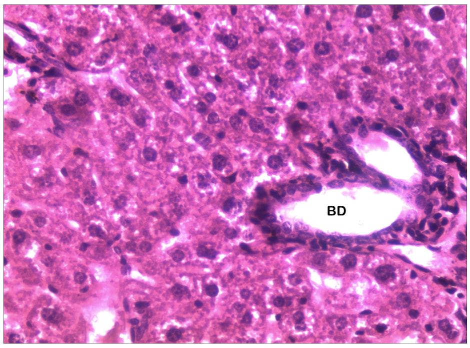

400x

B. PRK treated Mice

Lungs

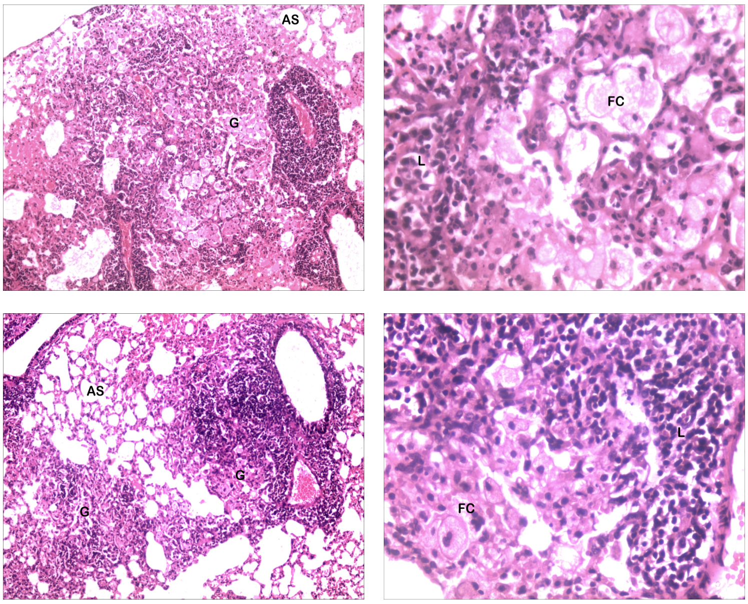

100x 400x

Spleen

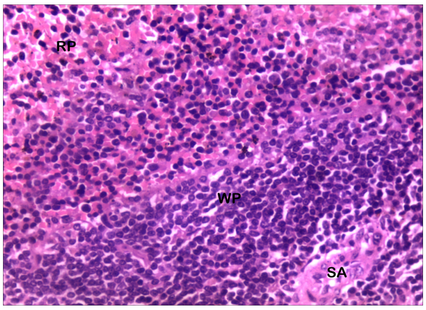

400x

Hepatic tissues

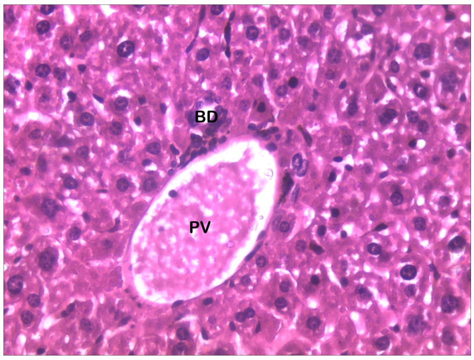

400x

Appendix Fig. S10. H&E staining of *Mtb* infected mice organs.

Mice infected with *Mtb* (H37Rv) were treated with **(A)** PBS (vehicle control) **(B)** PRK; sacrificed at day 24 shown here, and lungs, spleen and liver were isolated. The tissue samples were subjected to H&E staining and slides were sent for blind analysis. As observed, there was no spleen or liver associated toxicity in the PRK treated mice, as compared to control. (n=6)

Appendix Fig. S11

A.

Rifampicin treated Mice

Spleen

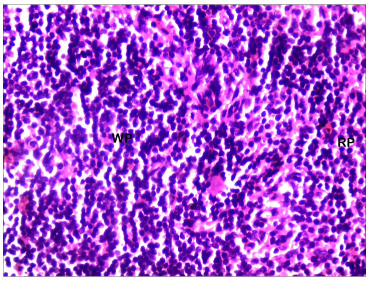

400x

Hepatic tissues

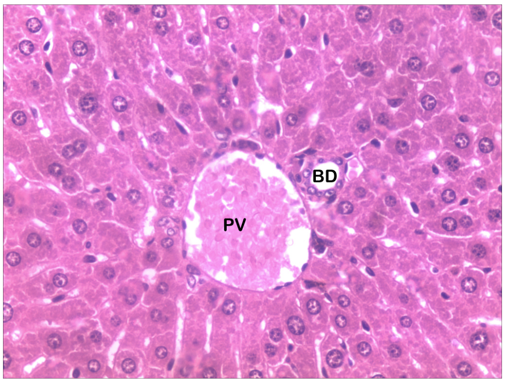

400x

Lungs

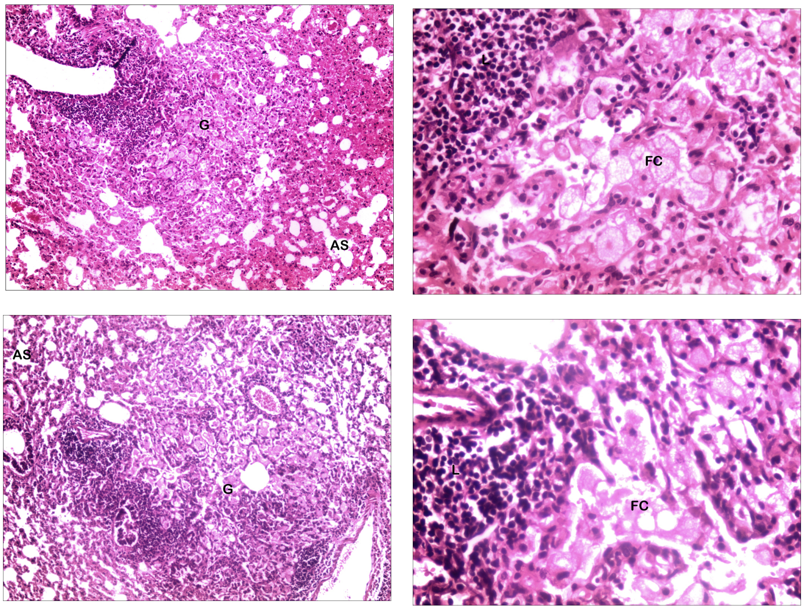

100x 400x

B.

PRK + Rif treated Mice

Spleen

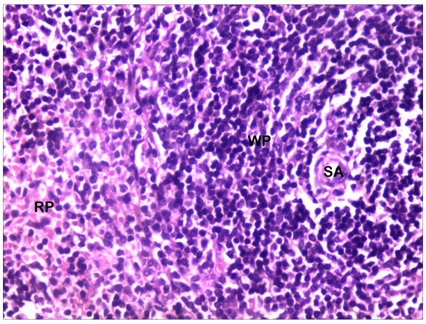

400x

Hepatic tissues

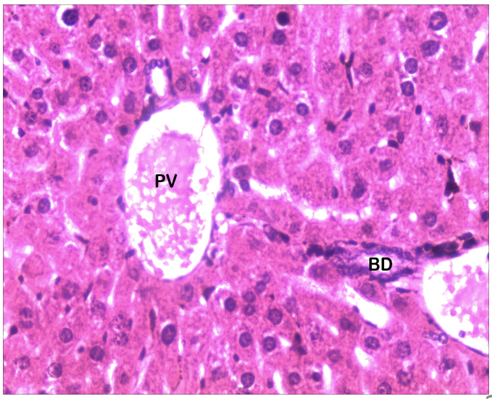

400x

Lungs

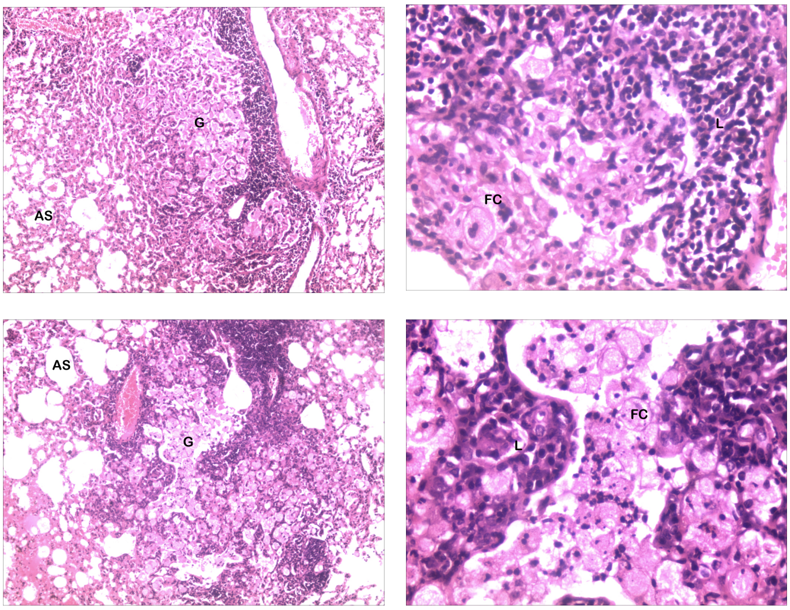

100x 400x

Appendix Fig. S11. H&E staining of *Mtb* infected mice organs.

Mice infected with *Mtb* (H37Rv) were treated with **(A)** Rifampicin (Rif) **(B)** Rif + PRK, sacrificed at day 24 shown here and lungs, spleen and liver were isolated. The tissue samples were subjected to H&E staining and slides were sent for blind analysis. As observed, there was no significant spleen and liver associated toxicity in the Rif + PRK treated mice, as compared to Rif only. (n=6)

Appendix materials and methods:

Mammalian cells, bacterial cultures, and infection:

Bacterial strains used in this study are *Mtb* H37Rv and the field isolates (MDR strains) Jal 2287, Jal 2261 (kind gift from Dr. Amit Singh, IISc, Bangalore). Bacteria were grown in Middlebrook 7H9 broth (BD 271310) supplemented with 10% Middlebrook OADC (BD 211886), 0.1% Glycerol and 0.1% Tween 80 until the mid-log phase (OD_{600} of 0.8). The human monocytic cell line THP-1 and mouse macrophage cell line, RAW 264.7, were maintained in an atmosphere containing 5% CO_2 at 37°C in the culture medium recommended by ATCC. THP-1 cells were cultured in RPMI-1640 media (Gibco- 22400-089) and Raw 264.7 cells were cultured in DMEM media (Gibco- 11965-092), both supplemented with 10% FBS (Gibco- 10437036). THP-1 monocytes were differentiated into macrophages by a 24 h treatment with 10 ng/ml phorbol 12-myristate 13-acetate (PMA; Sigma-Aldrich cat no. P1585, batch. no. SLBL1806V). Cells were rested for 3 days following chemical differentiation to ensure that they reverted to a resting phenotype before infection.

Pranlukast- Tocris biosciences (cat. no. 3026, Batch no. 1A/170719)

Sorafenib- AK Scientific (cat. no. V0124, Batch no. 3L41I)

N-acetyl Ornithine- Sigma (cat. no. A3626, Batch no. BCBJ2225V)

Rifampicin- Sigma (cat. no. R7382, Batch no. SLBD2314V)

Isoniazid- Sigma (cat. no. I3377, Batch no. SLBC3024V)

Ethambutol- Sigma (cat. no. E4630, Batch no. MKBR0925V)

Cloning and protein purification:

*Mt*ArgJ gene (Rv1653 gene) from the genome of *Mycobacterium tuberculosis* H37Rv strain was cloned into pET22b vector with a c-terminal six Histidine-tag using the standard cloning protocol (Molecular Cloning: A Laboratory Manual. Joseph

Sambrook, David W. Russell, 2001). E coli (BL21-DE3) cells were transformed with the cloned plasmid and a colony was inoculated for primary culture in Luria Broth supplemented with Ampicilline (100µg/ml) and allowed to grow till the OD reached 0.8. Secondary culture was then inoculated with 1% primary culture and allowed to grow at 37°C till OD reached 0.6. The culture was given a 15-min cold shock at 4°C and then protein production was induced by adding IPTG (0.5mM) and incubated at 16°C for 14 hours. The culture was centrifuged (5000rpm) post induction and cells were washed with PBS. The pellet was dissolved in lysis buffer (20mM Tris buffer, 200mM NaCl, 2mM PMSF and 1mM beta-mercapto-ethanol) and sonicated at 28% amplitude for 30 minutes (2sec on and 6sec off). The sonicated mixture was centrifuged (14000 rpm) for 45 minutes and the supernatant was incubated with Ni-NTA beads for affinity purification on an end-on rocker for 1 hour. Flow-through was discarded and beads were washed with 100ml of Wash buffer 1 (Lysis buffer+10mM imidazole) and 50mL of wash buffer 2 (Lysis buffer+20mM imidazole). Elution fractions of 2 ml each were collected (EB1-50mM imidazole-repeat twice, EB2-100mM imidazole-repeat twice, EB3-200mM imidazole, EB4-400mM imidazole). The elutions with pure protein were dialyze against 20mM tris (pH7.2) and 100mM NaCl with 10% glycerol. To determine the concentration of active *Mt*ArgJ we densitometrically determined the percentage of cleaved fragments out of total protein from SDS-PAGE profile and then calculated the concentration of that fraction, which contributes to the active protein in the solution.

Macrophage infection with *Mtb* and CFU analysis:

RAW 264.7 macrophages and differentiated THP-1 cells seeded separately at 10^4 cells per well in 96-well plates were infected with *Mtb* H37Rv at the moi of 2 (macrophage:bacteria = 1:2) and incubated for 4 hours at 37°C in 5% CO_2. Extracellular bacteria were removed by washing twice with PBS and incubating with

200μg/ml Amikacin (Himedia- cat. no. CMS644) for 1 hour followed by two additional rounds of PBS washing. Cells were incubated with varying concentration of inhibitors for documented amount of time. For combination experiments, Rifampicin (MIC- 0.156 μg/ml), Isoniazid (MIC- 0.125μg/ml), and Ethambutol (MIC- 12μg/ml) were mixed at the concentration of 40 ng/ml, 30 ng/ml and 1.5 μg/ml respectively and the cocktail was used to treat *Mtb* (H37Rv) as well as *Mtb* infected macrophages.

The cells were incubated with the cocktail and the desired concentration of PRK/SRB in CO_2 incubator (5%) at 37°C and then washed with PBS thrice prior to being lysed by adding 0.06% SDS solution in 7H9 media to harvest the internalized *Mtb.* The harvested *Mtb* solution is then washed with PBS and plated on *Difco* 7H11 agar solid media supplemented with 10% ADC at three different dilutions and incubated at 37°C incubator for 21 days. The colonies were counted and CFU was determined for each experimental condition. All the experiments involving pathogenic strains of *Mtb* were performed inside an isolated Biosafety level-3 (BSL-3) facility at Centre for Infectious Disease Research (CIDR) IISc, Bangalore.

RNA extraction, cDNA synthesis and Q-PCR:

Macrophages were infected with *Mtb* as mentioned above, treated with the drug for 12 hours and total RNA from the macrophages were extracted by the RNeasy Mini Kit (Qiagen) and cDNA was synthesized by the standard protocol using random hexamers and M-MuLV Reverse Transcriptase from New England Biolabs. This was followed by the Q-PCR amplification of the subsequent transcripts from the synthesized cDNA samples using SYBR green Q-PCR mix (BIORAD).

Computational methods:

Binding site prediction:

Binding pockets in *Mycobacterium tuberculosis* ornithine acetyltransferase (MtArgJ) were predicted using a web server MetaPocket 2.0 (http://projects.biotec.tu-dresden.de/metapocket) (Huang, 2009). For this, 3D X-ray crystallography structure of MtArgJ (PDB Id: 3IT6) was obtained from RCSB PDB (http://www.rcsb.org). Metapocket 2.0 uses a consensus method, eight different methods were deployed to predict the binding sites with high accuracy. The results of the top sites predicted from each method are presented. Additionally, CASTp webserver was employed to calculate the area volume of the different binding clefts (Dundas *et al*, 2006).

Virtual drug library screening protocol

Protein preparation protocol

Virtual ligand screening for the identification of best hit compounds was conducted using the X-ray coordinates of Ornithine Acetyltransferase from *M. tuberculosis* resolved at 2.4Å (MtArgJ; PDB ID: 3IT6) (Sankaranarayanan *et al*, 2010) and obtained from RCSB Protein Data Bank (www.rcsb.org). Prior to docking, substrates were removed from the pocket, enzyme coordinates were corrected for missing side chains, checked for unnatural amino acids, non-standard atom types and atom occupancy factor using SwissPDB viewer (Guex & Peitsch, 1997).

Drug library

For this study, FDA-approved compound library was considered for drug repositioning and was downloaded from DrugBank (http://www.drugbank.ca/) **(Wishart, 2006)**. The multi-molecule file obtained in sdf format was split and converted to 3D coordinates, formal charges and hydrogen atoms were added and saved in Mol2 format using MolConverter module of Marvin (Version 16.8.29.0, 2015, ChemAxon (http://www.chemaxon.com)). To this dataset, 8-Anilino-1-naphthalenesulfonic acid

(ANS) was added, which served as a reference compound to determine the cut-off during ligand screening.

Docking simulations

All docking calculations were performed using Dock 6 (v6.6) (**http://dock.compbio.ucsf.edu**) **(Allen *et al*, 2015)**. The protein receptor was processed using Dock Prep module of Chimera using AMBER parm99 partial charges and then output in Mol2 format **(Pettersen *et al*, 2004)**. Active site was identified and prepared by selecting spheres at a distance from 1-10Å from the ANS. All the input files required to define the negative image of the binding site were prepared to superpose the ligands using the programs present in the DOCK distribution. Ligands were protonated and assigned AM1-BCC charges and then docked. Standard flexible docking protocol was employed to sample internal degrees of freedom of the ligands. The important parameters are: minimum of five atoms were treated as rigid, the maximum number of anchor orientations attempted is set to 1000, a maximum of 1000 orientation were retained using a pruning clustering coefficient of 100 combining conformer rank and RMSD. A simplex minimizer was employed with score convergence set to 0.1 kcal/mol and translational, rotational, and torsional stepsizes for the simplex minimizer were set to 1.0 Å, 0.1p radians, and 10.0, respectively. A single round of simplex minimizer constituted a maximum of 500 iterations for the anchor and 500 iterations for the partially grown molecule. A pruning score cut-off was maintained at 100 kcal/mol to reject the conformers greater than the cut-off after energy minimization. Grid scoring based on the intermolecular non-bonded terms viz., van der Waals (VDW) for steric and electrostatic interactions was computed using AMBER force field ff99 to identify the best orientation of each ligand. Finally, the best

scored conformer was retained for examination and further refinement and Amber based scoring.

Scoring functions and filtering criteria

(i) Bivariate histogram-based partitioning method

The first filtering criterion applied was based on the receptor-ligand non-bonded energy terms computed from the standard flexible docking protocol. 2D bivariate histogram-based partitioning method was applied to obtain ligands possessing significant non-covalent interaction energies comparable with ANS. For this, bins with a minimum of five ligands were chosen as cut-off followed by culling out those with van der Waals and electrostatic energies greater than ANS.

(ii) Amber GB⁄SA based rescoring and filtering

Amber scoring function implements molecular mechanics implicit solvent simulation based on generalized Born/surface area (GB/SA) continuum model on the topmost poses for further refinement. The individual ligand, the individual receptor, and the ligand-receptor complex were minimized, subjected to MD simulation allowing flexibility in both ligand and receptor within a distance of 8 Å and more minimized to calculate the binding free energies. The score is computed as $E_{binding} = E_{Complex} - (E_{Receptor} + E_{Ligand})$, where the energy terms included bond, angle, dihedral terms, Coulomb's Law and the Lennard-Jones potential for the ligand, receptor, and complex along with the electrostatic and non-polar part of solvation energy calculated using GB/SA (Lang *et al*, 2009).

(iii) Receptor-ligand complex based filtering

The third filtering criterion was based on a set of parameters defining the receptor-ligand complex formation, viz., electrostatic binding free energies, ligand strain, gap

volume, change in total solvent accessible surface area and violation of Lipinski's rule of five.

(a) Violation of Lipinski's rule of five: Compounds were initially grouped based on the violation of Lipinski's rule of five (Lipinski *et al*, 1997) computed using the chemviz plugin of cytoscape (http://www.cgl.ucsf.edu/cytoscape/chemViz/index.shtml). Ligands were categorized into three groups, for instance, compounds that fail no rule, failed only one rule and more than one rule into 0, 1 and 2 groups, respectively.

(b) Binding free energies: Electrostatic component of binding free energies (ΔΔGpol) were computed using the thermodynamics cycle of Adaptive Poisson−Boltzmann Solver (APBS) software package (Baker *et al*, 2001). All the parameters were set to default. For computing Coulombic binding free energies, the protein and ligands were transferred from solvent to vacuum to compute the difference in energies $-\Delta G_{pol\ protein}$ and $-\Delta G_{pol\ ligand}$, respectively. Coulombic energies for the complex formed by combining protein and ligand in vacuum are determined as ΔECoulombic = ECoulombic complex −ECoulombic protein− ECoulombic ligand and the corresponding energy cost of the solvate complex was determined as ΔGpol complex. Finally, the electrostatic component of binding free energies ($\Delta\Delta G_{pol}$) was computed as

$$\Delta\Delta G_{pol} = \Delta G_{pol}^{complex} - \Delta G_{pol}^{protein} - \Delta G_{pol}^{ligand} + \Delta E_{Coulombic}$$

(c) Ligand conformational strain energy: Conformational energies of ligands in free and bound state in solution were estimated using APBS software package. The energy penalty associated with a deformation occurred due to protein interaction of ligand is defined as ligand strain energy and computed from the difference in its bound and free conformation. The cut-off to cull the ligands with high deformation was determined as 8.3 kJ/mol (2 kcal/mol) (Butler *et al*, 2009; Perola & Charifson, 2004).

(d) Gap Volume Index: Gap volume index is a measure for surface complementarity obtained by normalizing gap volume with their interface area (Zhu *et al*, 2006). Gap regions in MtArgJ-ligand complexes were computed by fitting gap spheres of minimum 1.0 Å and maximum 5.0 Å radius between all pairs of atoms and reducing the spheres until all clashes with other atoms are avoided using the SURFNET program (Laskowski, 1995). The grid separation was set to 2.0 Å. Interface area defined as one half of the ΔSASA (change in SASA upon complexation of MtArgJ and the ligands) was calculated using NACCESS program.

$$\text{Gap Volume Index} = \frac{\text{Gap Volume}}{\text{Interface Area}}$$

Median-based partitioning method was employed to separate ligands showing best surface complementarity than others (Godden *et al*, 2002). A binary classification scheme was then deployed to divide the population into two equal subpopulations by assigning the ligands having a value below the median as “0” and value above the median as “0”.

(e) Change in Solvent accessible surface area: Change in total solvent accessible surface area (ΔSASA) upon binding was calculated from the differences between the complex and the sum of free molecules. For this, NACCESS program with a probe radius of 1.4 Å was used to determine the changes occurred in accessible surface area upon binding. Like in gap volume descriptor, Median-based partitioning method was applied to determine the cut-off value followed by binary classification scheme assigning the values above the cut-off as “0” and below the median as “1”.

(iv) ***Receptor-ligand non-covalent interactions based filtering***

Intermolecular non-covalent interactions were calculated using BINANA program to identify important binding characteristics like hydrogen bonds, hydrophobic contacts, salt bridges, and pi interactions (Durrant & McCammon, 2011).

Molecular Dynamics simulations

3D coordinates of MtArgJ deposited in RCSB PDB (PDB ID: 3IT6) was previously considered for virtual screening was also used for all-atom molecular dynamic simulations. Initially, free-MtArgJ coordinates were subjected to molecular dynamic simulations as implemented in GROMACS 5.1.1 (Abraham *et al*, 2015). System was prepared using CHARMM36 force field and TIP3P water model (Mackerell *et al*, 2004). The molecular system was energy minimized in vacuum for 1000 steps employing steepest descent algorithm and 500 steps on conjugate gradient minimization. Subsequently, periodic boundary conditions were defined by adjusting the boundaries of the cubic box by 10Å. Water and sodium ions were added to the unit cell to maintain overall charge neutrality. Once again, energy minimization was done for 5000 steps to stabilize the solvated systems. Position restrained and unrestrained MD simulations were carried out to equilibrate the solvated system at temperature 300K using Berendesen thermostat under 1 atm pressure using Parinello–Rahman pressure coupling bath. During simulations, all bonds were constrained using LINCS algorithm. Electrostatic calculations were accounted by particle-mesh ewald (PME) method with a cut-off distance for Coulomb and van der Waals interactions maintained at 1.4 nm. The final production simulations for each MtArgJ system in free form were simulated for 50ns. Subsequently, molecular systems of MtArgJ complexes generated with two N-acetyl ornithine molecules docked in the two substrate pockets (MtArgJ-NAO) and three inhibitors viz. *8*-Anilinonaphthalene-1-*sulfonic acid* (MtArgJ-ANS), pranlukast (MtArgJ-PRK) and sorafenib (MtArgJ-SRB) into the hydrophobic binding site and simulated for 50ns. The topological parameters for the substrate and inhibitor molecules were generated using SwissParam webserver (http://www.swissparam.ch/) (Zoete *et al*, 2011).

Appendix results and discussions:

Binding site prediction

Crystal structure of *Mycobacterium tuberculosis* ornithine acetyltransferase (PDB ID: 3IT6) obtained from Protein Data Bank is a 41 kDa dimer constituting 404 amino acids in each monomer (Sankaranarayanan *et al*, 2010) . The two active sites are formed by five loops that connected α5→β10, β8→β9, α6→β11, α4→β7, and β6→α3, whereas the large hydrophobic pocket is composed of residues contributed by both the monomers. The large interface pocket comprised of four loops flanking β4→α1, β8→β9, α5→β10, α9→β12, four helices – α5, α7, α8, α10 and one β15 strand contributed by each monomer (Appendix Fig. S1b-d). This well-defined pocket was found to be a large cleft of area 2019.7Å^2 and volume 3104.8Å^3. However, we noticed that loops flanking β8→β9 and α5→β10 were positioned at the interface of this cavity and both of the substrate binding pockets.

Virtual drug library screening protocol

Receptor-ligand interactions:

Non-covalent interactions for 43 ligands were computed and tabulated (Appendix table S2). Hydrogen bonds, hydrophobic bonds, cation-pi and salt bridges were quantified for the MtArgJ-ligand complexes (Appendix Fig. S2.C, D). We noticed that hydrogen bonds ranged between 0 - 9, hydrophobic bonds ranged between 8 – 115, cation-pi interactions ranged between 0 – 2 and salt bridges ranged between 0 – 3. Appendix Fig. S2.E shows the docked conformations of all the 43 ligands within the allosteric cavity on *Mt*ArgJ.

Negative validation of in-silico screening strategy:

For negative validation of our *in-silico* screening strategy, we tested 10 compounds from the non-selected groups (Appendix table S4) by TLC based enzymatic assay. None of them showed any inhibition in the *Mt*ArgJ enzymatic activity (data not shown). This further confirmed the robustness of our methodology.

MOLECULAR DYNAMICS SIMULATIONS

Stability of *Mt*ArgJ in free and complex form:

Stability of MtArgJ in free and complexed states were quantified based on RMSDs and RMSFs computed for the simulation trajectories. RMSDs of backbone of MtArgJ in unbound and bound form with substrate and inhibitors were shown in a plot of RMSD versus time (Appendix Fig. S3.A). The average backbone-RMSD of free MtArgJ was noted to be 0.17±0.02 Å and substrate bound MtArgJ (MtArgJ-NAO) was calculated as 0.16±0.05 Å, whereas MtArgJ bound to ANS (MtArgJ-ANS), pranlukast (MtArgJ-PRK) and sorafenib (MtArgJ-SRB) exhibited backbone RMSDs of 0.14±0.02 Å, 0.14±0.03 Å and 0.18±0.06 Å, respectively. After 25ns of MD simulations, MtArgJ bound to pranlukast exhibited a sudden blip in the backbone-RMSD rising to about 0.25Å but reached an equilibrium. However, MtArgJ in complex with ANS and sorafenib exhibited gradual increase in RMSDs.

RMS fluctuations of residues in active sites and inhibitor binding pocket

RMSF of residues lining the active sites and the interfacial residues between the active site and allosteric site were computed. Fluctuations of the eight sustrate interacting residues in monomer-1 (active site formed by chains A and B) are shown in Appendix Fig. SB.(i-iii). We noticed that the RMSF of all the residues were around 4Å. Even the seven residues contributing to the hydrophobicity in the active site indicated that RMSFs of Met193, Leu194, Ala195 and Pro196 increased in the monomer-1 upon binding to inhibitors. Likewise, the interface loop residues between active site of

monomer-1 and the inhibitor binding site showed RMSF of about 4Å for eight residues except for Ser238, which exhibited RMSF > 3.5 nm (Appendix Fig. S3-B.iii). We observed that fluctuations of Ser238 in MtArgJ complexed with inhibitors decreased relative to substrate bound MtArgJ, whereas reverse is true for other residues although the change in RMSF was found to be negligible. Similarly, RMSF of active site residues and interface loop of monomer-2 (active site formed by chains C and D) were computed and represented in Appendix Fig. S3(iv-vi). Ser238 of monomer-2 showed higher RMSF compared to other interface loop residues as in monomer-1. However, the computed hydrophobic residues in the active site of monomer-2 indicated that RMSF of Gly190 and Pro191 increased, Gly192 and Met193 showed no significant difference and Leu194, Ala195 and Pro196 decreased in MtArgJ complexed with inhibitors. Upon examining the RMSF of inhibitor binding residues, we found that except for Asp234 of chain B, RMSFs decreased for Gln305, Arg308 and Ser310 of chain B and D.

Distances between residues involved in intra protein and protein-substrate interactions

The critical interactions of active site residues with substrate (NAO) were measured as a function of time. The interatomic distances were computed between Thr127($O^{\gamma1}$) and Thr200(O), Gly128(N^{α}) and Thr200(O), Thr200($O^{\gamma1}$) and Ser238(O^{γ}) and Ser238(O^{γ}) and Asp241($O^{\delta1}$) in MtArgJ-NAO complex and the distance for modes from probability distribution function were measured to be 0.41 Å, 0.71 Å,0.28Å and 0.44 Å, respectively for monomer-1 and 0.52 Å, 0.79 Å,0.27Å and 0.44 Å, respectively in monomer-2. Appendix Fig. S4.(A, B) represents the plot for distances among the active site residues in monomer-1 and 2, respectively. We also calculated the distances between the active site residues viz. Thr127, Gly128 and

Thr200 and N-acetyl ornithine in both the monomers, which were found to be 0.27 Å, 0.28 Å and 0.30 Å respectively for both the monomers. Further, in the inhibitor bound state of *Mt*ArgJ-NAO complex, except for the interatomic distance between Ser238(O^{γ}) and Asp241($O^{\delta 1}$), all other distances increased. Appendix Fig. S5 (A, B) shows the distances between the active site residues and substrate molecule (N-acetyl Ornithine) in monomer-1 of MtArgJ in complex with inhibitors, computed as a function of time.

Hydrogen bond interactions of MtArgJ with ANS:

Hydrogen bond interactions existing for more than 5% of the MD simulation time were considered. ANS was found to interact with Gln305 of both B and D chains of both the monomers and Arg308 of chain D (Appendix Fig. S.6-A). Appendix table S5 and S6 summarizes the interactions computed between the active site residues and substrate molecules for both the monomers MtArgJ-NAO complex and compared with inhibitor bound complexes.

To further our study, we computed the interatomic distances of crucial active site residues involved in enzyme catalysis (Thr200 and Ser238) and oxyanion hole stabilization (Thr127 and Gly128) (Sankaranarayanan *et al*, 2010). In inhibitor bound MtArgJ, the interatomic distances between the carbonyl oxygen of catalytic residue Thr200(O) has increased with the side chain oxygen of Thr127 ($O^{\gamma 1}$) and backbone amine of Gly128(N^{α}) resulting in the deformation of oxyanion hole. Similarly, the distance between Thr200($O^{\gamma 1}$) and Ser238(O^{γ}) has increased in inhibitor bound MtArgJ, whereas the distance between Ser238(O^{γ}) and Asp241($O^{\delta 1}$) has decreased forming a strong hydrogen bond when compared to MtArgJ-NAO complex. This indicates that Ser238 was withdrawn form hydrogen bonding with Thr200 and thus destabilized the acyl-enzyme complex. Furthermore, the substrate interactions of side

chain oxygen of Thr127 and main chain nitrogen of Gly128 has increased indicating perturbation of the negative charge on the oxyanion hole. In MtArgJ-NAO complex, the distance between the N-acetyl ornithine and $O^{\gamma 1}$ of Thr200 was noted to be 3Å, ideal for nucleophilic attack, but upon inhibitor interaction, the hydrogen bond was perturbed implying positional disruption of Thr200 and thus enzyme catalysis.

Appendix Table S1: Table showing the codes and their significance in filtering of ligands based on the five receptor-ligand complex parameters

**Code**	**Filters applied**				
	**Violation of Lipinski's rule of five**	**Binding Free energies (kcal/mol)**	**Ligand Strain Energy (kJ/mol)**	**% Change in Solvent accessible surface area ΔSASA**	**Gap Index (GI)**
**0**	Zero rule violated	≤ -1000	≤ 8.3	≥ 55.86%	≤ 76.63
**1**	One rule violated	between -1000 to -500	> 8.3	> 55.86%	> 76.63
**2**	≥ 1 rule violated	> -500	-	-	-

Appendix Table S2: List of 43 ligands, distributed in four groups with non-covalent interactions with the *Mt*ArgJ allosteric pocket

Cluster	Ligands	Hydrogen Bonds	Hydrophobic Bonds	Cation-Pi	Salt Bridge	Total contacts
**A (00000)**	Arformoterol	5	63	0	0	68
	Avanafil	0	67	0	0	67
	Cisapride	7	15	2	0	24
	Delaviridine	4	45	0	0	49
	Doxazosin	2	27	0	0	29
	Ixabepilone	1	22	0	0	23
	Moricizine	4	54	0	0	58
	Ticagrelor	2	49	1	0	52
**B (10000)**	Almitrine	0	47	0	0	47
	Amprenavir	7	34	0	1	42
	Dalfopristine	3	42	0	0	45
	Darunavir	7	8	0	0	15
	Dofetilide	4	56	1	0	61
	Methotrexate	7	36	0	3	46
	Mupirocin	3	115	0	0	118
**C (01000)**	Amsacrine	3	36	0	0	39
	Azetreonam	4	34	0	1	39
	Benzthiazide	3	39	0	0	42
	Carbenicillin	2	40	0	1	43
	Erlotinib	5	67	0	0	72
	Felodipin	3	17	0	0	20
	Fluvastatin	1	25	0	1	27
	Gefitinib	2	37	2	0	41
	Hydroxychloroquin	2	50	0	0	52
	Moxifloxacin	3	34	0	0	37
	Nimodipine	2	44	0	0	46
	Nisoldipine	1	17	0	0	18
	Paliperidine	2	21	0	0	23
	Pentamidine	4	60	0	1	65
	Risoperidine	2	12	0	0	14
	Rosiglitazone	1	35	1	0	37
	Rosuvastatin	3	32	0	0	35
	Sitaxentan	3	41	0	0	44
	Sulfadoxin	4	47	0	0	51
	Tauroursodeoxy-cholic Acid	5	13	0	0	18

	Trimetrexate	9	46	0	1	56
**D (11000)**	Astemizole	0	57	2	1	60
	Cabergoline	2	58	1	0	61
	Maravrioc	1	69	2	0	72
	Pitavastatin	2	40	0	0	42
	Pranlukast	6	70	1	0	77
	Pravastatin	1	27	0	0	28
	Sorafenib	3	20	0	0	23

Appendix Table S3: List of compounds screened *in vitro* from the selected groups to test the inhibition of *Mt*ArgJ enzyme activity:

Sr. no.	Name	Sr. no.	Name	Sr. no.	Name
1	Amprenavir	6	Dalfopristine	11	Moxifloxacin
2	Astemizole	7	Darunavir	12	Pentamidine
3	Azetreonam	8	Delaviridine	13	Pranlukast
4	Benzthiazide	9	Dofetilide	14	Sorafenib
5	Cisapride	10	Methotrexate	15	Trimetrexate

Appendix Table S4: List of compounds screened (*in vitro*) from the non-selected group for validation of *in silico* screening strategy:

Sr no.	Name	Sr No.	Name
1	Nilotinib	6	Dasatinib
2	Tipranavir	7	Thiamine
3	Montelukast	8	Aspartame
4	Zafirlukast	9	Biotin
5	ANS	10	Ciprofloxacin

Appendix Table S5: Hydrogen bond interactions between active site residues and substrate

Active site pockets	Hydrogen bond interactions	MtArgJ-NAO (% existence)	MtArgJ-ANS (% existence)	MtArgJ-PRK (% existence)	MtArgJ-SRB (% existence)
**Monomer - 1**	Thr127($O^{\gamma1}$) – NAO(O)	5.4	-	-	-
	Thr127($O^{\gamma1}$) – NAO(O1)	78.01	-	4.85	-
	Gly128(N^{α}) – NAO(O1)	97.05	-	9.52	-
	Thr166($O^{\gamma1}$) – NAO(O)	97.55	37.19	15.26	97.53
	Thr166($O^{\gamma1}$) – NAO(O1)	-	28.07	79.56	-
	Thr166($O^{\gamma1}$) – NAO(O2)	-	-	-	17.22
	Lys189(N^{ζ}) – NAO(O)	30.31	87.99	56.11	-
	Lys189(N^{ζ}) – NAO(O1)	-	13.00	-	-
	Lys189(N^{ζ}) – NAO(O2)	-	7.37	-	-
	Thr200(N^{α}) – NAO(O)	-	50.78	63.94	-
	Thr200(N^{α}) – NAO(O1)	-	-	-	91.62
	Thr200(N^{α}) – NAO(O2)	89.71	51.97	32.24	93.91
	Thr200($O^{\gamma1}$) – NAO(N1)	21.73	32.34	-	-
	Thr200($O^{\gamma1}$) – NAO(O)	-	5.09	-	42.91
	Thr200($O^{\gamma1}$) – NAO(O1)	26.61	-	6.38	12.04
	Thr200($O^{\gamma1}$) – NAO(O2)	32.55	39.28	36.08	52.35
	Gly192(O) – NAO(NE)	20.02	-	-	-
	Glu280($O^{\varepsilon1}$) – NAO(NE)	37.60	51.41	38.67	53.09

	Glu280(O$^{\varepsilon2}$) – NAO(NE)	50.89	40.46	56.88	43.92
	Glu398(O$^{\varepsilon1}$) – NAO(NE)	-	11.78	-	-
	Glu398(O$^{\varepsilon2}$) – NAO(NE)	-	11.49	-	-
	Asn399(O$^{\delta1}$) – NAO(NE)	39.03	30.29	39.10	43.24
	Ser404(O^{OT1}) – NAO(N1)	-	-	23.83	40.48
	Ser404(O^{OT2}) – NAO(N1)	-	-	-	72.79
**Monomer - 2**	Thr127(O$^{\gamma1}$) – NAO(O)	-	-	-	5.539
	Thr127(O$^{\gamma1}$) – NAO(O1)	96.45	-	99.16	6.70
	Gly128(N$^{\alpha}$) – NAO(O1)	95.06	-	95.2	6.75
	Thr166(O$^{\gamma1}$) – NAO(O)	18.56	99.69	99.08	34.19
	Thr166(O$^{\gamma1}$) – NAO(O)	-	-	-	50.47
	Thr167(O$^{\gamma1}$) – NAO(NE)	11.58	-	-	-
	Lys189(N$^{\zeta}$) – NAO(O)	-	-	-	11.41
	Lys189(N$^{\zeta}$) – NAO(O2)	55.27	-	-	5.15
	Thr200(N$^{\alpha}$) – NAO(O)	9.88	-	-	23.17
	Thr200(N$^{\alpha}$) – NAO(O1)	-	93.82	-	11.52
	Thr200(N$^{\alpha}$) – NAO(O2)	82.06	97.35	98.93	48.40
	Thr200(O$^{\gamma1}$) – NAO(O1)	-	-	-	16.34
	Thr200(O$^{\gamma1}$) – NAO(O1)	-	14.83	-	-
	Thr200(O$^{\gamma1}$) – NAO(O2)	22.56	-	-	22.74
	Glu280(O$^{\varepsilon1}$) – NAO(NE)	9.16	47.72	-	52.92

	Glu280($O^{\varepsilon2}$) – NAO(NE)	9.03	54.33	98.44	42.67
	Glu398($O^{\varepsilon2}$) – NAO(NE)	-	-	-	6.43
	Asn399($O^{\delta1}$) – NAO(NE)	13.87	78.19	34.64	34.68
	Ser404(O^{γ}) – NAO(NE)	44.63	-	-	20.56
	Ser404(O^{OT2}) – NAO(N1)	-	98.61	-	23.16

Appendix Table S6: Hydrogen bond interactions between allosteric site residues and the inhibitors

MtArgJ-NAO-Inhibitor complex	Chain (Monomer)	Hydrogen bond interactions	% Existence
**8-Anilino-1-naphthalenesulfonic acid (ANS)**	B (Monomer – 1)	Gln305($N^{\varepsilon 2}$) – ANS(O3)	16.26
	D (Monomer – 2)	Gln305($N^{\varepsilon 2}$) – ANS(O1)	9.213
		Arg308(NH1) – ANS(O2)	33.078
**Pranlukast (PRK)**	B (Monomer – 1)	Asp234(O) – PRK(N3)	8.01
		Gln305($N^{\varepsilon 2}$) – PRK(O2)	5.54
		Gln305($N^{\varepsilon 2}$) – PRK(N1)	6.46
		Ser310(N) – PRK(O4)	34.96
		Ser310(O^{γ}) – PRK(O4)	18.46
	D (Monomer – 2)	Ser310(N) – PRK(N2)	11.55
		Ser310(N) – PRK(N4)	6.78
		Ser310(O^{γ}) – PRK(N4)	6.82
		Ser310(O^{γ}) – PRK(N5)	6.20
**Sorafenib (SRB)**	B (Monomer – 1)	Arg308(NH1) – SRB(O1)	27.64
	D (Monomer – 2)	Gln305($O^{\varepsilon 1}$) – SRB(N2)	14.55
		Arg308(O) – SRB(N1)	7.57

Appendix Table S7: Exact p-values and 'n' for all the statistical calculations

Figure no.	Experiment	p-value	n
**Figure 2. d**	Control vs. ANS (0.5mM)	0.1	4
	Control vs. ANS (5mM)	0.02	4
**Figure 5. f**	RHE vs. RH+PRK (0.01μg/ml)	0.0009	3
	RHE vs. RH+SRB (1μg/ml)	0.00007	3
**Figure 5. g**	RHE vs. RH+SRB (0.01μg/ml)	0.04	3
	RHE vs. RH+SRB (1μg/ml)	0.001	3
**Figure 6. a**	SRB (5μg/ml) vs. PRK (0.5μg/ml)	0.001	3
	SRB (5μg/ml) vs. PRK (5μg/ml)	0.00009	3
**Figure 6. b**	SRB (5μg/ml) vs. PRK (0.5μg/ml)	0.01	3
	SRB (5μg/ml) vs. PRK (5μg/ml)	0.0001	3
**Figure 6. c**	RHE vs. RH+SRB (0.5μg/ml)	0.004	3
	RHE vs. RH+PRK (0.5μg/ml)	0.00007	3
**Figure 6. d**	RHE vs. RH+SRB (0.5μg/ml)	0.007	3
	RHE vs. RH+PRK (0.5μg/ml)	0.0001	3
**Figure 6. f**	(THP1) *Mtb* vs. 32 hr	0.0002	3
	(RAW 264.7) *Mtb* vs. 32 hr	0.0001	3
**Figure 6. j**	(THP1) *Mtb* vs. PRK (1μM)	0.0004	3
	(THP1) *Mtb* vs. PRK (10μM)	0.0001	3
	(RAW 264.7) *Mtb* vs. PRK (1μM)	0.04	3
	RAW 264.7) *Mtb* vs. PRK (10μM)	0.0002	3
**Figure 7. a**	DMSO vs. PRK (1μg/ml)	0.0006	3
	PRK (1μg/ml) vs. PRK+Arg (1mM)	0.0040	3
**Figure 7. b**	DMSO vs. PRK (0.1μg/ml)	0.040	3
	DMSO vs. PRK (0.5μg/ml)	0.001	3
**Figure 7. c**	Uninfected vs. infected:		
	CysLTR1	0.00006	3
	5-LO	0.00007	3
	FLAP	0.0002	3
	Cox-2	0.007	3
	MCP-1	0.006	3
**Figure 7. d**	DMSO vs. PRK (5μg/ml)	0.00009	3
**Figure 7. e**	DMSO vs. PRK (5μg/ml)	0.00007	3
**Figure 7. f**	DMSO vs. PRK (5μg/ml)	0.004	3
**Figure 7. g**	DMSO vs. PRK (5μg/ml)	0.0003	3
**Figure 7. h**	DMSO vs. PRK (5μg/ml)	0.0007	3
**Figure 8. f**	PBS vs. PRK (day-12)	0.01	6
	PBS vs. PRK (day-24)	0.003	6
**Figure 8. g**	Rif. vs. Rif. +PRK (day-12)	0.0002	6

	Rif. vs. Rif. +PRK (day-24)	0.0001	6
**Figure 8. h**	Control vs. PRK	0.0007	6
	Control vs Rif.	0.0004	6
	Rif. vs. PRK+Rif.	0.002	6

References:

Abraham MJ, Murtola T, Schulz R, Páll S, Smith JC, Hess B & Lindahl E (2015) GROMACS: High performance molecular simulations through multi-level parallelism from laptops to supercomputers. *SoftwareX* **1-2:** 19–25

Allen WJ, Balius TE, Mukherjee S, Brozell SR, Moustakas DT, Lang PT, Case DA, Kuntz ID & Rizzo RC (2015) DOCK 6: Impact of new features and current docking performance. *Journal of Computational Chemistry* **36:** 1132–1156

Baker NA, Sept D, Joseph S, Holst MJ & McCammon JA (2001) Electrostatics of nanosystems: Application to microtubules and the ribosome. *Proceedings of the National Academy of Sciences* **98:** 10037–10041

Butler KT, Luque FJ & Barril X (2009) Toward accurate relative energy predictions of the bioactive conformation of drugs. *Journal of Computational Chemistry* **30:** 601–610

Dundas J, Ouyang Z, Tseng J, Binkowski A, Turpaz Y & Liang J (2006) CASTp: computed atlas of surface topography of proteins with structural and topographical mapping of functionally annotated residues. *Nucleic Acids Research* **34:** W116–W118

Durrant JD & McCammon JA (2011) BINANA: A novel algorithm for ligand-binding characterization. *Journal of Molecular Graphics and Modelling* **29:** 888–893

Godden JW, Xue L, Kitchen DB, Stahura FL, Schermerhorn EJ & Bajorath J (2002) Median Partitioning: A Novel Method for the Selection of Representative Subsets from Large Compound Pools. *Journal of Chemical Information and Computer Sciences* **42:** 885–893

Guex N & Peitsch MC (1997) SWISS-MODEL and the Swiss-Pdb Viewer: An environment for comparative protein modeling. *Electrophoresis* **18:** 2714–2723

Huang B (2009) MetaPocket: A Meta Approach to Improve Protein Ligand Binding Site Prediction. *OMICS: A Journal of Integrative Biology* **13:** 325–330

Lang PT, Brozell SR, Mukherjee S, Pettersen EF, Meng EC, Thomas V, Rizzo RC, Case DA, James TL & Kuntz ID (2009) DOCK 6: Combining techniques to model RNA-small molecule complexes. *RNA* **15:** 1219–1230

Laskowski RA (1995) SURFNET: A program for visualizing molecular surfaces, cavities, and intermolecular interactions. *Journal of Molecular Graphics* **13:** 323–330

Mackerell AD Jr., Feig M & Brooks CL III (2004) Extending the treatment of backbone energetics in protein force fields: Limitations of gas-phase quantum mechanics in reproducing protein conformational distributions in molecular dynamics simulations. *Journal of Computational Chemistry* **25:** 1400–1415

Perola E & Charifson PS (2004) Conformational Analysis of Drug-Like Molecules Bound to Proteins: An Extensive Study of Ligand Reorganization upon Binding. *Journal of Medicinal Chemistry* **47:** 2499–2510

Pettersen EF, Goddard TD, Huang CC, Couch GS, Greenblatt DM, Meng EC & Ferrin TE (2004) UCSF Chimera?A visualization system for exploratory research and analysis. *Journal of Computational Chemistry* **25:** 1605–1612

Sambrook J, Russell DW (2001) Molecular Cloning: A Laboratory Manual. *The Quarterly Review of Biology* **76:** 348–349

Wishart DS (2006) DrugBank: a comprehensive resource for in silico drug discovery and exploration. *Nucleic Acids Research* **34:** D668–D672

Zhu H, Domingues F, Sommer L & Lengauer T (2006) NOXclass: prediction of protein-protein interaction types. *BMC Bioinformatics* **7:** 27

Zoete V, Cuendet MA, Grosdidier A & Michielin O (2011) SwissParam: A fast force field generation tool for small organic molecules. *Journal of Computational Chemistry* **32:** 2359–2368
